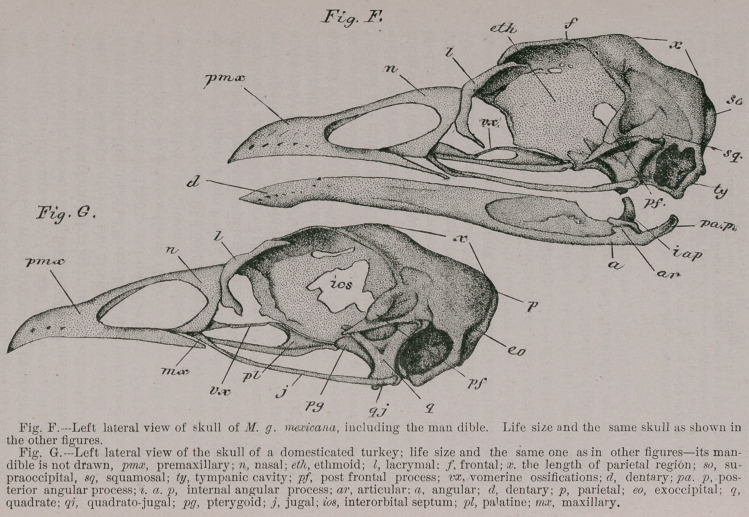# A Critical Comparison of a Series of Skulls of the Wild and Domesticated Turkey

**Published:** 1887-07

**Authors:** R. W. Shufeldt

**Affiliations:** Captain, Medical Corps, U. S. Army


### THE JOURNAL

OF

### COMPARATIVE MEDICINE AND SURGERY.

VOL. VIII. JULY, 1887. No. 3.

### ORIGINAL COMMUNICATIONS.

### ART. XX. A CRITICAL COMPARISON OF A SERIES OF SKULLS OF THE WILD AND DOMESTICATED TURKEYS.

(*Melegris gallopavo mexicana and M. domestica.*)

BY R. W. SHUFELDT, C. M. Z. S

*Captain, Medical Corps, U. S. Army.*

As one would surely have predicted beforehand, Mr. Darwin, when he came to compare series of skulls of the numerous varieties of the domesticated fowls, with the skull of *G. bankiva*, and similarly, skulls of a number of species of tame ducks with the wild duck, found some very striking differences among them. So far as I have been able to ascertain, however, he never compared the skulls of the tame and wild turkeys; and so far as his comparisons of fowls and ducks are concerned, I believe they are principally intended to show the great variation that has taken place in these parts, and the marked departures from the wild type in the case of the fowl and duck, respectively.

Indeed, it would be difficult I imagine, unless one could secure skulls from an entire line of fowls showing the gradual changes in them as they descended from the parent wild stock, to demonstrate anything else. The same remark applies, of course, to the ducks. I am not aware that any such a series has ever been made, with the view of pointing out these interminate variations, as they must have occurred in some of the breeds. It would not be difficult, however, to picture to our minds the shading differences that would take place in the skulls in a line of fowls extending between *G. bankiva*, and, for instance, a white-crested Polish Cock.

With respect to the turkeys we have some very interesting data to start from, and of such a character, I think, that when taken in connection with the facts that I intend to present in this paper, it will lend some additional light to certain phases of this question.

In the first place, our ornithologists now recognize two well-defined species of wild turkeys in the avifauna of the United States, viz : *M. gallopavo*, and *M. g. mexicana:* then there are in this country alone, several very well-marked varieties of the domesticated turkey. So there seems to be no reason but that by careful selection and breeding we might not in time have quite as many varieties of turkeys as we now have of chickens, and presenting the same extraordinary differences in form and plumage.

Further, to quote quite extensively from Mr. Darwin's "Animals and Plants under Domestication," (Vol I, pp. 352-355), and omitting the authorities from whom he derived some of his information, we find that: "It seems fairly well established by Mr. Gould, that the turkey, in accordance with the history of its first introduction, is descended from a wild Mexican species (*Meleagris mexicana*) which had been already domesticated by the natives before the discovery of America, and which differs specifically, as it is generally thought, from the common wild species of the United States."

"Some naturalists, however, think that these two forms should be ranked only as well-marked geographical races.

However this may be, the case deserves notice because in the United States wild male turkeys sometimes court the domestic hens, which are descended from the Mexican

Fig. A. Superior aspect, life size, of a skull of a wild turkey, *M. g. mexicana.*

Fig. B. The same view of a skull of the domesticated turkey, also life size. Drawn from adult specimens by the author, and mandibles removed in each case. *pmx*, premaxillary ; *pl*, palatine ; *mx*, maxillary ; *l*, lacrymal ; *f*, frontal ; *mx. p.*, maxillo-palatine ; *n.*, nasal ; *eth.*, ethmoid ; *ju.*, jugal ; *q j.*, quadrato-jugal ; *q.*, quadrate ; *so.*, supraoccipital ; *sq.*, squamosal ; *pf.*, postfrontal process.

form, and are generally received by them with great pleasure. Several accounts have likewise been published of young, reared in the United States from the eggs of the wild species, crossing and commingling with the common breed. In England, also, this same species has been kept in several parks; from two of which the Rev. W. D. Fox procured birds, and they crossed freely with the common domestic kind, and during many years afterwards, as he informs me, the turkeys in his neighborhood clearly showed traces of their crossed parentage * * * English turkeys are smaller than either wild form. They have not varied in any great degree ; but there are some breeds which can be distinguished, as Norfolks, Suffolks, Whites, and copper-colored (or Cambridge), all of which, if precluded from crossing with other breeds, propagate their kind only." Darwin then goes on in the same place to point out some of the marked characteristics of the other varieties, one or two of which were conspicuously crested. He concludes by saying that "In India the climate has apparently wrought a still greater change in the turkey, for it is described by Mr. Blyth as being much degenerated in size, 'utterly incapable of rising on the wing,' of a black color, and with the long pendulous appendages over the beak enormously developed."

With these facts before us, I conceived it would be of interest, if not of actual importance to compare a good series of selected skulls of *M. g. mexicana*, with a series of skulls of that domesticated form of the turkey which shows in its external characters evidences of being still closely affined to the wild stock. Then taking into consideration the number of years since this bird has been domesticated, I thought it might be possible to discover those definite characters in the skull that already showed a departure from the corresponding features in the skull of the wild turkeys. The latter are to be found in the forests within a mile of my present residence, and this gave me the opportunity, which I have availed myself of, of securing a fine series of the skulls of this form. Another series representing the variety of the tame turkey alluded to in the last paragraph, were collected for me in Chicago, by Mr. H. K. Coale, the president of the Ridgway Ornithological Club of that city ; and I am under great obligations to him for the evident care he took to select the proper kind of material. I prepared the skulls of both of these series myself, as the heads came to me in the flesh. Not being familiar with any name, as having previously been bestowed upon it by former authors, for this variety of the tame turkey, I have designated it for convenience sake, the *M. g. domestica.*

When we come to simply superficially compare the skull of one of these wild turkeys with the skull of one of the domesticated ones, we appreciate that same difference which we find upon a similar comparison to distinguish the skull of a cock *G. bankiva*, and any of the typically domesticated fowls. It seems to consist in a lightness, a pneumaticity, accompanied by a certain sharpness of the details of the skull, an angularity, if we may so express it, in the case of the wild bird, as contrasted with an evident thickness and density of the bone, together with a general mellowing down of its principal free edges, producing a certain lack of sharpness, in the case of the domesticated one.

Now to compare the details, I have chosen the skull, one from my series, of a fine adult male specimen of *M.g. mexicana*, it having all the features of a skull of a wild turkey well exemplified. This skull I have drawn life size in the figures illustrating this paper (Figs. A, C, D and F). With the same care I have selected for illustration one of the skulls of the series representing my tame turkeys, which seems to present all the salient characters seen in the skull of *M. g. domestica* (Figs. B, E and G). It was also an adult male specimen.

Viewing these two skulls upon their superior aspects, as shown in figures A and B, we find the form of the premaxillary bone essentially very much the same in both birds ; and, I fail to find any distinctive differences among them that have any claim to constancy. It will be noticed that the backward-extending supero-median nasal process of this bone retains throughout life its longitudinal division into two slips. Between the posterior extremities of these, in all specimens that I have examined, both wild and tame, it is possible to discern the unlying ethmoid (*eth*). Coming next to the nasal bones (*n*), we find that they also have pretty much the same shape and relation in the two skulls under consideration. I have always noticed, however, that in the skulls of wild turkeys, the *posterior borders* of the nasals indistinguishably fuse with the adjacent frontals, and in them this fronto-nasal region is more concaved than it is in the skulls of the domestic turkeys. There is one skull of a tame turkey in my series, and but one, that shows this absorption of the fronto-nasal suture. But this skull also exhibits other features that partake more or less of the characteristics of the skull of a wild turkey.

I am inclined to think that it will be found though, that the persistency of this suture in the skull of adult tame turkeys, marks one of those differences that will eventually become one of its established distinctive characters. And here it will be as well to remark that the fusing of those bones of the skull that commonly have the sutures among them obliterated in adult life, takes place, as a rule, at a much later date in domesticated turkeys, than it does in the wild birds. It is not an uncommon thing to find the bifrontal suture present in old barn-yard gobblers. What I have said in regard to the premaxillary and nasal bones, is well shown in figures A and B; we may also see there the usual form assumed by the lacrymal (*l*), which latter is a largely developed element of the skull in all turkeys. In the vast majority of skulls of both species this bone articulates with the external free edge of the posterior moiety of the corresponding nasal; it may, however, in either species, slightly encroach upon the adjacent frontal bone. Its horizontal portion seems to be longer and more pointed in wild turkeys, than in the tame ones; we will probably find numbers of exceptions to this rule, however, but a still more constant character is to be found in the descending portion of the bone, which is evidently much longer, and more conspicuous in the former than it is in the latter species.

Passing now to the fronto-interorbital region, it is the rule so far as I have examined, that the transverse diameter here is manifestly greater in the wild turkey than it is in the domesticated bird; while, as I have already stated, the fore part of this region is more sunken in the former fowl. Posterior to the frontal area again, we find the parietal prominences better marked in wild turkeys than they are in tame ones.

There are two other well-marked and comparable characters upon this aspect of these skulls, but as they can be better appreciated upon the lateral view, I will defer their discussion until we come to consider that part of our subject.

Let us now pass to the posterior views of these skulls, as shown in figures D and E; and, beginning from the top, we observe the more prominent parietal prominences in the

Rear views, life size, of the skulls of the wild (Fig, D) and tame (Fig. E) turkeys. *f*, frontal bone; *p*, parietal; *pf*, postfrontal process. *qj*, quadrato-jugal; *q*, quadrate; *fm*, foramen magnum; and *o. c.*, occipital condyle. In both specimens the mandibles have been removed.

wild turkey, over the evenly-rounded, corresponding region of the domesticated one. The principal feature, however, to be taken into consideration upon this aspect of the skull is, what I please to call here, the *occipital area*. By the occipital area I mean that space so definitely circumscribed upon this face of the cranium by the bounding occipital ridge or line. In a great many birds the general form of this area, constitutes upon comparison a very good character. The rule here is, that in the tame turkey this area is decidedly more rounded than we ever find it in the wild one, although we occasionally observe in the former that it assumes the cordate outline which, so far as my researches carry me, is invariably the case in *M. g. mexicana.*

Little or no difference seems to distinguish the form of the occipital condyle among these fowls, for both in tame and wild turkeys, we find the notch at its middle point above to be deeply cleft in some cases, whereas in others it is barely perceptible.

The occipital bone as a whole is thicker and apparently denser in the tame turkey than it is in the wild one, but as to the relative size of the brain cavities, I would prefer to measure a much larger series of skulls than I now have at my disposal. I would say, though, that little if any change has taken place in this particular ; and to accurately decide upon this important point, at least a hundred skulls for either species should be carefully measured, averaged and compared. If this ever be undertaken I simply predict that the result will show that the average capacity of the brain cavity will be found to be rather larger in the wild turkey than it is in the tame one, contrary to the usual rule following domestication, I believe.

Upon lateral view of these two typical skulls we find for comparison but three points that demand our special consideration ; these are, *the arch of the superior margin of the orbit ; the depth of the parietal region ;* and, *the interorbital septum* (Figs. F and G).

First, as to the arch of the superior margin of the orbit, we find this more elevated, and, as it were, more convexed in the wild than it is in the tame turkey, where this arc is depressed, long and shallow, and but slightly raised above the plane of the frontal region.

Another very well marked character and one rarely departed from, is the depth of the parietal region ; what I mean by this is the distance measured on a median longitudinal line from the parietal prominences to the occipital ridge. This line is proportionately much shorter, and less horizontal in the wild turkey than it is in the domesticated one. By the aid of this character alone, I believe I could in a mixed collection of these two species of turkey, correctly pick out the skulls of the vast majority that belonged to either kind. This difference is indicated by $x$ in Figs. F and G.

As to the condition of the interorbital septum I would say that, in all the specimens of *M. g. mexicana*, which I have examined, this bony plate is entire and of considerable thickness. I have found this to be the case in but one instance in the series of skulls of the tame turkeys at my command, while in all the others of this latter species an irregular vacuity of some size exists in it (Fig. G, *ios*).

Before passing to the consideration of the characters at the base of the skull, it would be as well to state that the skulls of wild turkeys differ as a rule from each other but very little, and only to an extent due to the usual variance of each individual skull, whereas in a series of these specimens chosen from the domesticated turkey, we occasionally find a skull which in its several details more closely approaches the average skull of the wild bird.

Now at the base of the skull I fail to find any constant differences in the basitemporal area of the two series, or in the quadrates; or the infraorbital bars: the palatines; the basis, basisphenoidal rostrum; the maxillo-palatines; or in the under side of the premaxillary, this last bone having already been alluded to.

In the case of the *pterygoids*, however, I think it will as a rule be found, that in the wild species they are rather longer and slenderer than in the skull of the average tame turkey. Certainly it is so in the specimens before me. Although not showing any distinctive difference between the two varieties, for it appears to be equally well developed in both series. I have found the "vomerine ossifications" the most interesting features at the base of the skull of these turkeys.

According to the bibliography of Professor Elliott Coues, Owen published in 1837 in P. Z. S., a paper entitled a "Dissection of the Head of the Common Turkey (*Meleagris gallopavo*)" comparing it at the same time with the head of the *Cathartes aura*. This contribution is not available to me at the present writing, and I am unable to say whether

Professor Owen passed any remarks in it upon the *vomer* or not.* I am not familiar with any other memoirs devoted especially to the anatomy of the *Meleagris*, and passing over the many recent text-books and memoirs referring to fowls, by the Parkers, Huxley, Claus, Bell and others, I take the statement of the author of the article "Birds" in the ninth edition of the Encyclopædia Britannica as final authority on the subject, and he says that "In the *Gallinaces*, as in the desmognathous *Rapaces*, the vomer is single; in Pigeons and Sand-Grouse it is absent."

Now the facts presented below are based upon careful dissections of mine of the heads of eleven turkeys in the flesh, of (the two kinds under consideration), with the view of determining the condition of the vomer, alone. Some of these investigations were made upon the heads while they were absolutely fresh; others had been a short time in alcohol; while still others were either parboiled, or had been submitted to prolonged though careful maceration. In all the cases the structures were examined under a powerful lens. I found the usual plane of soft tissues extending from the entire anterior margin of the ethmoid to the posterior margin of the cartilaginous nasal septum. Now when a bird possesses a single vomer, it is in this median plane of tissue that it is found, and for the common fowl, Professor W. K. Parker says, in his invaluable little treatise on the Morphology of the Skull, that, "The maxillopalatine plates (*mx. p.*) are broader and reach nearly to the mid line, being separated partly by the nasal septum and partly by the small vomer, which is rounded in front, and split for a short distance behind. The forks of the vomer (*v.*) articulate with the inner and anterior points of the inner plates of the palatine bones, which lie side by side mesially, nearly concealing the rostrum," (pp. 246, 247). Only in one turkey,

*In forwarding my proof for correction, Dr. Conklin, the *Journal's* Editor, did me the very great favor in sending with it the MSS. copy he had made of Prof. Owen's article, here alluded to, for which I am exceedingly grateful. Upon reading this over I find nothing in it respecting the *vomer*, or these *vomerine ossifications*, dealing as it does principally with the distribution of the olfactory nerves in the Vulture and Turkey, with the view of throwing light upon the question of the power of smell in the former bird. R. W. S.

and that an old domesticated gobbler, did I find any semblance of such a medium vomer, and in that specimen it was exceedingly small, close to the ethmoid, and composed of bone of the most elementary character. The aperture which fulfills the office of the "posterior nares" in a bird occurs in this locality, and the free edges of it extend from the anterior inner points of the palatines, to the corresponding apex of the maxillo-palatine, on either side. In the vast majority of the turkey heads which I examined, a delicate rod of bone is found in the soft tissues composing these free edges. So it will be seen that these two little rods of bone (Fig. C. *vx.*) extend from the anterior inner points of the palatines to the posterior apices of the maxillo-palatines, one of them on either side. Now it is with these "anterior inner points of the palatines," that the *vomer* in a common fowl articulates, the bonelet extending forward, as already stated, as a diminutive median spine. This calls up some interesting questions, for say the little *semi-ossified* piece in the median plane of tissue—which I discovered only in one very old turkey, and in it, it did not fork behind and have the posterior extremities of the forks "articulate with the anterior inner points of the palatines,"—does not represent the vomer, but that these little fully ossified rods, that I have just described, do; then we certainly have a singular departure in the turkey for a gallinaceous type, from the usual order of things.

In a head of a wild turkey now before me, an old adult male, I found no median ossification at all, to represent a vomer, but on the contrary both of these little rods are present and thoroughly ossified. As I write about this condition in the turkeys, my mind naturally reverts to what has been held for the *Pici*, and the organization in them of the corresponding parts; it has been said, as we know, that they have double vomers in adult life—but this was disputed by Garrod, ("*Ibis*" 1872 pp. 357-360), who claimed to have found a median vomer for the woodpecker.

A careful comparison of the *mandibles* of these two series of turkey skulls, fails to reveal to me any contestants of characters that could be relied upon to distinguish those belonging to the wild ones from those of the domesticated variety.

I have also compared the *hyoid arches:* the *sclerotal plates* of the eyeballs; and other minor ossifications about the skull, and what I have just said in regard to the mandibles, applies with equal force to them, there are no reliable characters to distinguish them.

This brings my comparisons of these two series of skulls to a close, and I will here complete my paper by a brief recapitulation of the constant characters which, so far as I have been able to ascertain, seem to distinguish the skull of a wild turkey from that of a domesticated one, the latter being descended from domesticated stock of long standing, and as free as possible from any mixture with the wild types.

In drawing up this summary, I would have it distinctly understood that only the most constant differences have been selected, and exceptions even to these may occasionally be found among tame turkeys, where for some unknown cause, they seem, in certain individual cases, to revert again to the cranial structure of the wild species.

These selected characters, will, however, show the tendency of the changes that are taking place, and are apparently up to the present time, typified in the skull of the tame turkey which I have chosen in the figures to illustrate them. I take it that these changes are still in somewhat of a transitional stage, and that eventually tame turkeys will differ quite widely from the wild ones. And that this difference will become much greater and more rapidly brought about when the breeding and selection of turkeys is more carefully looked into, with the view of introducing certain improvements in them.

### ANALYTICAL SUMMARY.

1. As a rule, in adult specimens of *M. g. mexicana*, the posterior margins of the nasal bones indistinguishably fuse with the frontals; whereas, as a rule, in domesticated turkeys their sutural traces persist with great distinctness throughout life.

2. As a rule, in wild turkeys we find the cranio-frontal region more concaved, and wider across than it is in the tame varieties.

3. The parietal prominences are apt to be more evident in *M. g. mexicana* than they are in the vast majority of domesticated turkeys; and the median, longitudinal line measured from these to the nearest point of the occipital ridge is longer in the tame varieties than it is in the wild birds. Generally speaking, this latter character is very striking and rarely departed from.

4. The figure formed by the line which bounds the occipital area, is, as a rule, roughly semi-circular in a domesticated turkey, whereas in *M. g. mexicana* it is nearly always of a cordate outline, with the apex upwards. In the case of the tame turkeys I have found it to average one exception to this in every twelve birds; in the exception, the bounding line of the area made a cordate figure as in wild turkeys.

5. Among the domesticated turkeys, the interorbital septum almost invariably is pierced by a large irregular vacuity; as a rule, this osseous plate is entire in wild ones.

6. The descending process of a lacrymal bone is more apt to be longer in a wild turkey than in a tame one; and for the average the greater length is always in favor of the former species.

7. In *M. g. mexicana*, the arch of the superior margin of the orbit is more decided than it is in the tame turkey, where the arc formed by this line is shallowed, and not so elevated.

8. We find, as a rule, that the pterygoid bones are rather longer and more slender in wild turkeys than they are among the tame ones.

9. At the occipital region of the skull, the osseous structures are denser and thicker in the tame varieties of turkeys; and, as a whole, the skull is smoother, with its salient apophysis less pronounced in them than they are in the wild types. There is a certain delicacy and lightness, very difficult to describe, that stamps the skull of a wild turkey, and at once distinguishes it from any typical skull of a tame one.

10. I have predicted that the average size of the brain cavity will be found to be smaller and of a less capacity in a tame turkey than it is in the wild one. In the case of this class of the domesticated animals, this would seem to be no more than natural, for the domestication of the turkey has not been of such a nature as to develop its brain mass through the influences of a species of education ; its long contact with man has taught it nothing—quite the contrary, for the bird has been almost entirely relieved from the responsibility of using its wits to obtain its food, or to guard against danger to itself. These factors are still in operation in the case of the wild types, and the advance of civilization has tended to sharpen them.

From this point of view then, I would say, that mentally the average wild turkey is stronger than the average domesticated one, and I believe it will be found that in all these long years, the above influences have affected the size of the brain-mass for the latter species in the way above indicated, and perhaps it may be possible some day to appreciate this difference. Perhaps, too, there may have been also a slight tendency on the part of the brain of the wild turkey to increase in size, owing to the demands made upon its functions due to the influences of man's nearer approach, and the necessity of greater mental activity in consequence.

## Figures and Tables

**Fig. A. Fig. B. f1:**
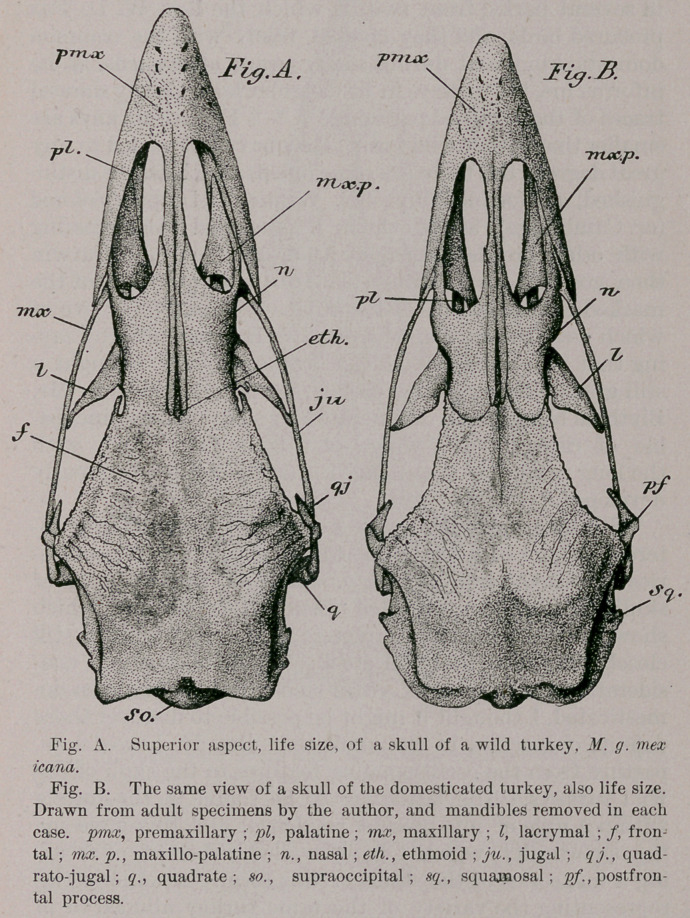


**Fig. C. f2:**
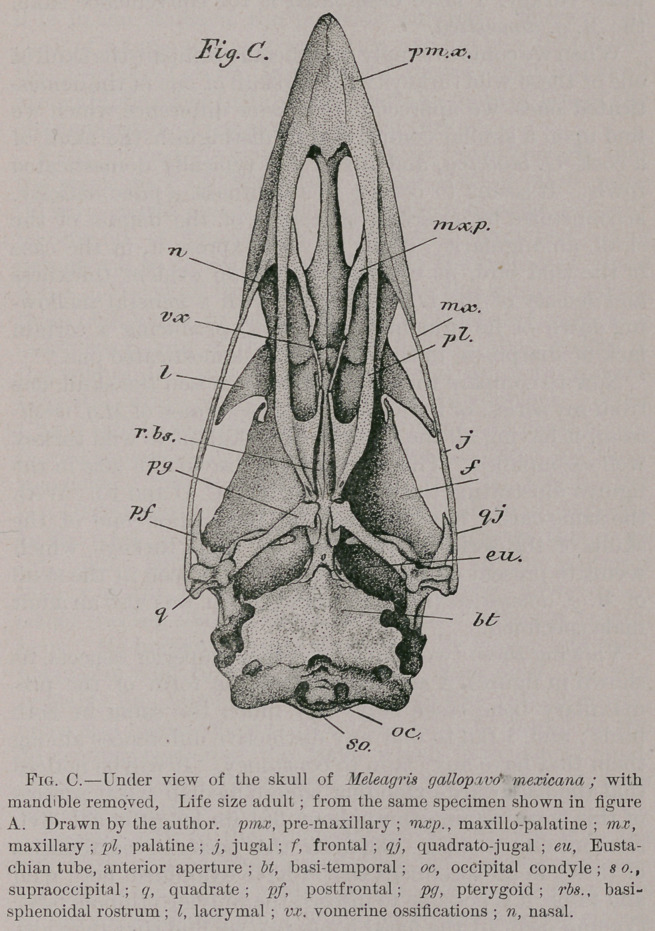


**Fig. D. Fig. E. f3:**
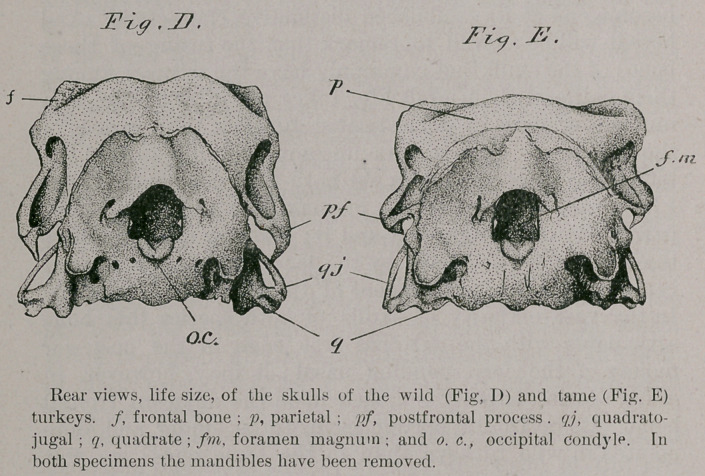


**Fig. F. Fig. G. f4:**